# Trajectories of Depressive Symptoms Among Web-Based Health Risk Assessment Participants

**DOI:** 10.2196/jmir.6480

**Published:** 2017-03-31

**Authors:** Richard Bedrosian, Matt Hawrilenko, Heather Cole-Lewis

**Affiliations:** ^1^ Johnson & Johnson Health & Wellness Solutions Johnson & Johnson New Brunswick, NJ United States; ^2^ Clark University Department of Psychology Worcester, MA United States

**Keywords:** depression, prodromal symptoms, health risk appraisal, health surveys, prevention

## Abstract

**Background:**

Health risk assessments (HRAs), which often screen for depressive symptoms, are administered to millions of employees and health plan members each year. HRA data provide an opportunity to examine longitudinal trends in depressive symptomatology, as researchers have done previously with other populations.

**Objective:**

The primary research questions were: (1) Can we observe longitudinal trajectories in HRA populations like those observed in other study samples? (2) Do HRA variables, which primarily reflect modifiable health risks, help us to identify predictors associated with these trajectories? (3) Can we make meaningful recommendations for population health management, applicable to HRA participants, based on predictors we identify?

**Methods:**

This study used growth mixture modeling (GMM) to examine longitudinal trends in depressive symptomatology among 22,963 participants in a Web-based HRA used by US employers and health plans. The HRA assessed modifiable health risks and variables such as stress, sleep, and quality of life.

**Results:**

Five classes were identified: A “minimal depression” class (63.91%, 14,676/22,963) whose scores were consistently low across time, a “low risk” class (19.89%, 4568/22,963) whose condition remained subthreshold, a “deteriorating” class (3.15%, 705/22,963) who began at subthreshold but approached severe depression by the end of the study, a “chronic” class (4.71%, 1081/22,963) who remained highly depressed over time, and a “remitting” class (8.42%, 1933/22,963) who had moderate depression to start, but crossed into minimal depression by the end. Among those with subthreshold symptoms, individuals who were male (*P*<.001) and older (*P*=.01) were less likely to show symptom deterioration, whereas current depression treatment (*P*<.001) and surprisingly, higher sleep quality (*P*<.001) were associated with increased probability of membership in the “deteriorating” class as compared with “low risk.” Among participants with greater symptomatology to start, those in the “severe” class tended to be younger than the “remitting” class (*P*<.001). Lower baseline sleep quality (*P*<.001), quality of life (*P*<.001), stress level (*P*<.001), and current treatment involvement (*P*<.001) were all predictive of membership in the “severe” class.

**Conclusions:**

The trajectories identified were consistent with trends in previous research. The results identified some key predictors: we discuss those that mirror prior studies and offer some hypotheses as to why others did not. The finding that 1 in 5 HRA participants with subthreshold symptoms deteriorated to the point of clinical distress during succeeding years underscores the need to learn more about such individuals. We offer additional recommendations for follow-up research, which should be designed to reflect changes in health plan demographics and HRA delivery platforms. In addition to utilizing additional variables such as cognitive style to refine predictive models, future research could also begin to test the impact of more aggressive outreach strategies aimed at participants who are likely to deteriorate or remain significantly depressed over time.

## Introduction

### Depressive Symptoms

Depressive symptoms play a significant role in determining the potential success of population health management efforts across the care continuum [1,2]. Comorbid depression increases risk at all levels of health, being associated with poor treatment adherence, impaired self-management skills, more frequent complications from illness, and poorer medical outcomes [1-3].

The negative impact of depressive symptoms is not confined to those who suffer from formal psychiatric disorders such as major depression. Research indicates that those with subthreshold depressive symptoms, who might not meet formal diagnostic criteria for depression at a given time, may still experience significant behavioral impairment and reduced quality of life, and can be at high risk for experiencing clinical levels of distress in the future [4,5].

Although all levels of depressive symptomatology have been found to increase risk of further difficulties, the use of statistical techniques such as growth mixture modeling (GMM) [6] has enabled researchers to map qualitatively distinct trajectories of depressive symptomatology over time and identify risk factors associated with them [7,8]. The output of GMM mapping is illustrated by a study of depression among low-income women recruited during pregnancy. Mora et al [7] found five distinct patterns: (1) always or chronic depressive symptomatology, (2) antepartum only, (3) postpartum, resolving after the first year postpartum, (4) late, present at 25 months postpartum, and (5) never having depressive symptomatology. Membership in the trajectory classes was influenced by several variables, including education, race, health behavior, and psychosocial characteristics such as ambivalence about pregnancy [7].

In theory, results of such longitudinal studies could be used to identify high-risk individuals and tailor preventative services for them. However, various types of longitudinal analyses conducted with different populations and using different measures report different trajectories associated with varying constellations of risk factors, suggesting that sample demographics, recruitment strategies, treatment context, and choice of measures affect the results of such investigations [7-9]. This variability in results suggests that risk profiles may vary across different populations. Consequently, to be effective, screening algorithms and preventative interventions may need to be tailored for specific populations (eg, pregnant women and elderly patients in primary care), based on data drawn from samples with similar characteristics.

### Health Risk Assessments

One unique group that may provide important insights and opportunities for intervention consists of adults who participate in mass screenings via Web-based health risk assessments (HRAs). HRAs are being administered to large segments of the population, particularly by employers. A recent report by the Kaiser Family Foundation found that 53% of large US employers (n≥200) and 23% of small employers (n<200) used HRAs with their employee populations, typically providing various incentives for participation [10]. Moreover, health plans may encourage or require members to take HRAs directly, or may create incentives for providers to administer them in clinical setting (ie, as part of the annual Medicare wellness visit). HRAs typically assess modifiable health risks, often including items covering depression, stress, and other behavioral health topics.

Because they are typically disseminated by employers rather than in clinical settings, HRAs constitute a unique channel for health screening, representing an opportunity to reach a different population, at different points in the disease continuum, as compared with patients being assessed in traditional health care settings. Employees and spouses are often incentivized on a yearly basis to take HRAs, thus providing longitudinal data on participants, both those with subthreshold symptoms and those with more significant distress.

This study used GMM [6] to examine longitudinal trends in depressive symptomatology among participants in an HRA used by employers and health plans in the United States. The primary research questions were the following: (1) Can we observe longitudinal trajectories in the HRA population like those that have been observed in other study samples? (2) Do HRA variables, which primarily reflect modifiable health risks, help us to identify predictors associated with these trajectories? (3) Can we make meaningful recommendations for population health management, applicable to HRA participants, based on the predictors we identify?

## Methods

### Participants

The sample initially consisted of deidentified data from 91,852 unique adult participants who completed the HealthMedia SUCCEED HRA [11]. We analyzed data only from those who took the HRA two or more times, as at least two assessment points are necessary to contribute information to growth trajectories, resulting in 22,963 participants in the final sample. Individuals participated in the HRA (at no cost to them) through employers or health care plans deploying the HRA as part of their population health offerings or health benefit structure. Table 1 presents the demographic characteristics of the sample.

**Table 1:** Sample characteristics (N=22,963).

Variable		Present sample^a^, n (%)
**Gender (n=22,907)**		
	Female	15,337 (66.95)
	Male	7570 (33.05)
**Age (n=22,912)**		
	18-24	576 (2.51)
	25-34	4333 (18.91)
	35-44	5130 (22.39)
	45-54	6543 (28.56)
	55-64	5690 (24.83)
	65 and older	640 (2.79)
**Ethnicity (n=22,812)**		
	Asian	469 (2.06)
	Black	3628 (15.90)
	Hawaiian	9 (0.04)
	Hispanic	1308 (5.73)
	Multiracial	209 (0.92)
	Native American	74 (0.32)
	Pacific Islander	39 (0.17)
	White	16,857 (73.90)
	Other	219 (0.96)
**Marital status (n=22,963)**		
	Single	2917 (12.70)
	Dating	1261 (5.49)
	Married	15,056 (65.57)
	Divorced	3284 (14.30)
	Widowed	445 (1.94)
**Education (n=22,798)**		
	Some high school	343 (1.50)
	High school graduate	3623 (15.89)
	Some college	6809 (29.87)
	College graduate	12,023 (52.74)

^a^Sample characteristics are based on raw, not multiply imputed data.

### Measures

All variables for this study were extracted from participant HRA responses. The HRA was voluntary: participants were informed that their responses would be aggregated for data analyses and customer reporting, but would not be individually shared with their health plan or employer. The HRA was administered in a Web format in nearly all instances, but was available in paper format as well.

Risk-related questions were typically derived from various validated scales, including the Cohen Perceived Stress Scale (PSS) [12], the Center for Epidemiological Studies Depression Scale (CES-D) [13-15] along with validated single-item measures of quality of life and health [16,17]. A modified version of the Work Productivity Activity Impairment (WPAI) questionnaire was used to measure worksite productivity impairment [18], a supplemental distal outcome measure for the study. To minimize the impact of transient acute sickness on productivity, WPAI questions referred to the past 4 weeks (rather than the past 7 days). The WPAI yields an estimate of total productivity impairment due to health, based on the combination of absenteeism and presenteeism. All predictor variables were measured at baseline. Data from this HRA have been extensively analyzed previously for other purposes [11,19].

#### Dependent Variable

The 10-item true-false version of the CES-D [13-15] was used to assess depressive symptoms. The score was computed as a sum of 10 items. CES-D scores ranged from 0 to 10 with higher scores indicating more depressive symptoms. The reliability in this sample was acceptable (Cronbach alpha=.76) and mean scores were like other nonclinical populations using a score of 4 as the cut-point for clinical significance [18,19].

### Analytic Strategy

The goal of this analysis was to identify subgroups of HRA participants with distinct depression trajectories and identify predictors that make useful discriminations between these subgroups. We used GMM [6] to accomplish this objective. The following elements are described below: (1) three key analytic decisions made before fitting the growth mixture model, (2) model-building and class enumeration strategy, (3) the procedure for including predictors and distal outcomes of trajectory class membership, and (4) handling of missing data.

#### Analytic Decisions Before Growth Modeling

We narrowed our time horizon to 15 months, the tail end of the time period at which participants completed their “one year” assessments. Longer-term follow-up data were too sparse to provide generalizable findings.

To account for varying lengths of time between observations, we discretized time, segmenting it into 5 waves based on the patterning of responses: baseline, 0.5-3 months, 3-6 months, 6-9 months, and 9-15 months. The number and temporal width of buckets were chosen after examining the patterning of responses, with the aim of balancing granularity with adequate covariance coverage. That is, we wanted buckets of time that were narrow enough to pool participant data into the same time point, but large enough to capture enough participants to have adequate overlap between time points for the model to be empirically identified. We chose the bucketing strategy because it was the most amenable option for generating nonlinear trajectories, which we anticipated would reflect the episodic nature of depression [20]. Other strategies (eg, multilevel) of handling nonequidistant assessments were not desirable because they are limited to modeling smooth, polynomial forms of time and would likely require more assessments per individual than offered by this dataset.

Because these data were skewed with a strong floor effect, we modeled depression as an ordered categorical variable. Growth mixture models are highly sensitive to distributional assumptions, and violating these assumptions can yield inaccurate results [21]. Discretizing the measure mitigated this problem by removing distributional and linearity assumptions. Previous research identified a cutoff score of 4 and above to indicate the presence of depression [14,15]. To increase granularity, we included two additional cutoffs to make a total of 4 categories. Scores of 0 and 1 were considered indicative of minimal depression, 2-3 subthreshold, 4-6 moderate, and 7-10 severe depression. We used a latent response variable specification, which models the observed outcome as a discretized form of an underlying continuous latent response variable [22]. This specification generates thresholds where the continuous latent variable maps onto the observed ordinal outcome. For our 4 categories of depression, latent response variable values below threshold 1 map to minimal depression, values between thresholds 1 and 2 map to subthreshold depression, values between thresholds 2 and 3 map to moderate depression, and values above threshold 3 map to severe depression.

#### Mixture Models and Class Enumeration

To model nonlinearity, we used freeloading growth curves. Factor loadings for baseline and endpoint were fixed at 0 and 1, respectively, and loadings for the remaining time points were freely estimated. We followed the class enumeration process and reporting guidelines outlined by Masyn [23], using a variety of fit statistics to guide model selection. Because the Bayesian information criterion (BIC) and consistent Akaike information criterion (CAIC) sometimes asymptote rather than peak, we used elbow plots to assess relative benefits of selecting a model with a large number of classes.

We were interested in identifying predictors that statistically significantly differentiated between latent classes where distinctions were of substantive interest, and distal consequences of latent class membership whose means varied across class. To develop a parsimonious prediction model, we initially screened candidate predictors by including single predictors as auxiliary variables to preview their relationship with the latent class variable, using an alpha criterion of *P*<.01. Once variables were screened into the prediction model, no trimming was performed. For the final model, variables were added stepwise to understand incremental changes. For both predictors and the distal outcome, we accounted for measurement error in the latent class structure using the manual 3-step approach described by Asparouhov and Muthén [24]. Continuous predictors, excluding age and sleep hours, were divided by their standard deviations to enhance interpretability.

#### Missing Data Strategy

This study had the benefit of a large N, but the drawback of sparse reporting between baseline and 1 year. Although there were a smaller mean number of assessments per individual than typically seen in growth curve models, the large N enabled us to generate subgroup trajectories using the information between baseline and 1 year, because there was adequate covariance coverage between all time buckets to identify the model. Coverage between adjacent time points was low, ranging from 1.2% to 16.6%, with N postbaseline ranging from 16.45% (3778/22,968; 6-9 months) to 46.00% (10,565/22,968; 9-15 months). Given the low covariance coverage, generalizability of findings is contingent on the validity of our assumptions about missing data. Therefore, understanding any patterning present in missing data is critical for assessing generalizability.

We identified three types of mechanisms that drove missingness in this analysis.

The first was our bucketing strategy. We chose the number of buckets post hoc, so the more thinly we sliced the buckets, the larger number of buckets and consequently, the more “missing” observations we would have. Using 5 buckets yielded substantial proportions of wave-by-wave missingness, given that participants averaged 2.10 responses over the course of follow-up, with only 9% of participants completing assessments at 3 or more waves. Missingness driven purely by design decisions and not participant characteristics meets criteria for missing at random. We used maximum likelihood estimation with robust standard errors, which produces unbiased estimates under conditions of missing at random, meaning that estimates are unbiased if missingness is either random or related only to variables that are included in the model.

The second mechanism was driven by the number of observations from each participant. The possibility that participants who provided more observations might have different characteristics than those who provided fewer data points could potentially bias estimates. To assess this mechanism, we dummy coded the number of assessments completed by each participant into 2 groups, participants with 2 versus participants with 3+ assessments, and compared them using logistic regression. We included all demographic and predictor variables in the study as covariates. We performed the analysis on the overall number of time points, and because it was possible that risk factors influenced the timing of completing an HRA, we also performed it wave-by-wave. By explicitly modeling this missingness mechanism, we could then include any significant predictors in the final model that determined class structure. By incorporating this information related to missingness in the model, maximum likelihood estimation would ensure estimates were unbiased by the mechanisms we identified.

The third issue was missing individual predictor variables. The proportion of this missingness was generally low, with the three largest proportions being hours of sleep (12.89%, 2961/22,968), physical activity (10.14%, 2328/22,968), and alcohol use (5.29%, 1214/22,968). Because these variables were exogenous, the maximum likelihood estimator did not contribute to their estimation. We generated 10 multiply imputed datasets (imputing only the X variables) using information from all study variables.

All analyses were conducted in Mplus 7.3 (Muthen & Muthen) [25].

## Results

### Baseline Comparisons

We compared individuals included and excluded from the study on baseline variables. Due to the large sample size, many statistically significant differences emerged, but all effect sizes were below the cutoff traditionally deemed a “very small effect” (*d*=.10). The largest differences were the following: Individuals in the excluded group had higher depression scores at baseline (Cohen *d*=.08), lower alcohol use (*d*=.08), and higher health quality (*d*=.04). Individuals included in the study were somewhat more likely to be married (65.57% vs 61.89%). Broadly, these suggest that individuals completing more HRAs had slightly poorer health and more health risk factors. Because all variables were included in the final model, these between-group differences did not bias model estimates.

### Class Enumeration

We found a 5-class model to provide the best blend of fit, parsimony, and interpretability. Fit statistics are presented in Table 2. We explored freeing within-class intercept and slope variances, but freeing either resulted in empirical under-identification and/or unstable solutions. Even across many random starts that used information from previous models to aid in convergence, likelihood values did not replicate, suggesting that the freeloading factor loadings consumed much of the variability within these data. Therefore, we only included models in the class enumeration process where within-class variances were fixed at 0. Fit improved at each model tested through 6 classes, and the 7-class model did not generate a stable solution. All fit criteria clearly favored the 5-class model over the 4-class model. Although the 6-class model had incrementally better fit than the 5-class model—and likelihood ratio tests also favored the 6-class model—the decreases in the BIC, CAIC, and approximate weight of evidence (lower values imply better fit) were markedly lower than between the 4- and 5-class models, suggesting the additive explanatory power of the sixth class was low. This 6-class model also showed evidence of class splitting, meaning that one class from the 5-class model was split into two qualitatively similar classes in the 6-class model. Additionally, only a very small proportion of likelihood values replicated, decreasing our confidence in the validity of the 6-class model. For this reason, we did not include it as a finalist in our candidate models or calculate any of the Bayesian statistics for comparative fit. We used the approximate correct model probability (cmP ^_A_), which is an approximation that a given model is correct out of a set of observed models, to compare the 4 and 5-class models; along with the other fit statistics, it strongly favored the 5-class model. Entropy for the 5-class model was .95, meaning the posterior classification of individuals into latent classes was fairly precise with individuals relatively cleanly separated between classes.

**Table 2:** Fit statistics for growth mixture models.

Model	# parameters	Loglikelihood	BIC^a^	CAIC^b^	AWE^c^	Bayes factor (*k* vs *k* +1)	cmP_A_^d^	
1-class^e^	8	−36,256	72,593	72,601	72,697	<.01	–	
2-class	14	−37,384	74,909	74,923	75,092	<.01	–	
3-class	20	−35,123	70,448	70,468	70,709	<.01	–	
4-class	26	−34,386	69,033	69,059	69,372	<.01	0	
5-class	32	−33,424	67,170	67,202	67,587	–	1	
6-class	38	−32,975	66,332	66,370	66,828	–	–	
7-class	Empirically underidentified							

^a^BIC: Bayesian information criterion.

^b^CAIC: consistent Akaike information criterion.

^c^AWE: approximate weight of evidence.

^d^cmP_A_: Probability that model is true among set of all models being compared.

^e^The intercept was not constrained to 0 in the 1 class model, allowing it to serve as a better benchmark. Values for the Lo-Mendell-Rubin and Bootstrapped Lo-Mendell-Rubin likelihood ratio tests are not included because they were all significant at *P*<.001 and not useful in distinguishing between models.

**Table 3:** Parameters for 5-class growth mixture model. The class proportions are based on model-estimated data, and differ slightly than those based on modal class assignment.

Parameters		Minimal (63.66%)	Low risk (18.65%)	Deteriorating (4.69%)	Remitting (8.05%)	Chronic (4.96%)
Intercept		−51.05	−38.14	−42.31	−30.35	−27.64
Slope		6.12	−5.14	12.56	−12.63	−0.98
**Factor loadings**						
	Baseline	0.00^a^	0.00^a^	0.00^a^	0.00^a^	0.00^a^
	0.5-3 months	−14.61	0.36	0.35	0.07	−1.51
	3-6 months	−16.52	0.26	0.09^b^	0.08	−1.24
	6-9 months	0.73	0.70	0.54	0.18	−1.17
	9-15 months	1.00^a^	1.00^a^	1.00^a^	1.00^a^	1.00^a^
**Thresholds**						
	Minimal	−43.01	All thresholds constrained constant across classes			
	Subthreshold	−35.02				
	Moderate	−27.90				
	Severe	–				

^a^Denotes parameters constrained constant across latent classes.

^b^This parameter is significant at *P*=.02. All other parameters are significant at *P*<.001.

**Figure 1:**
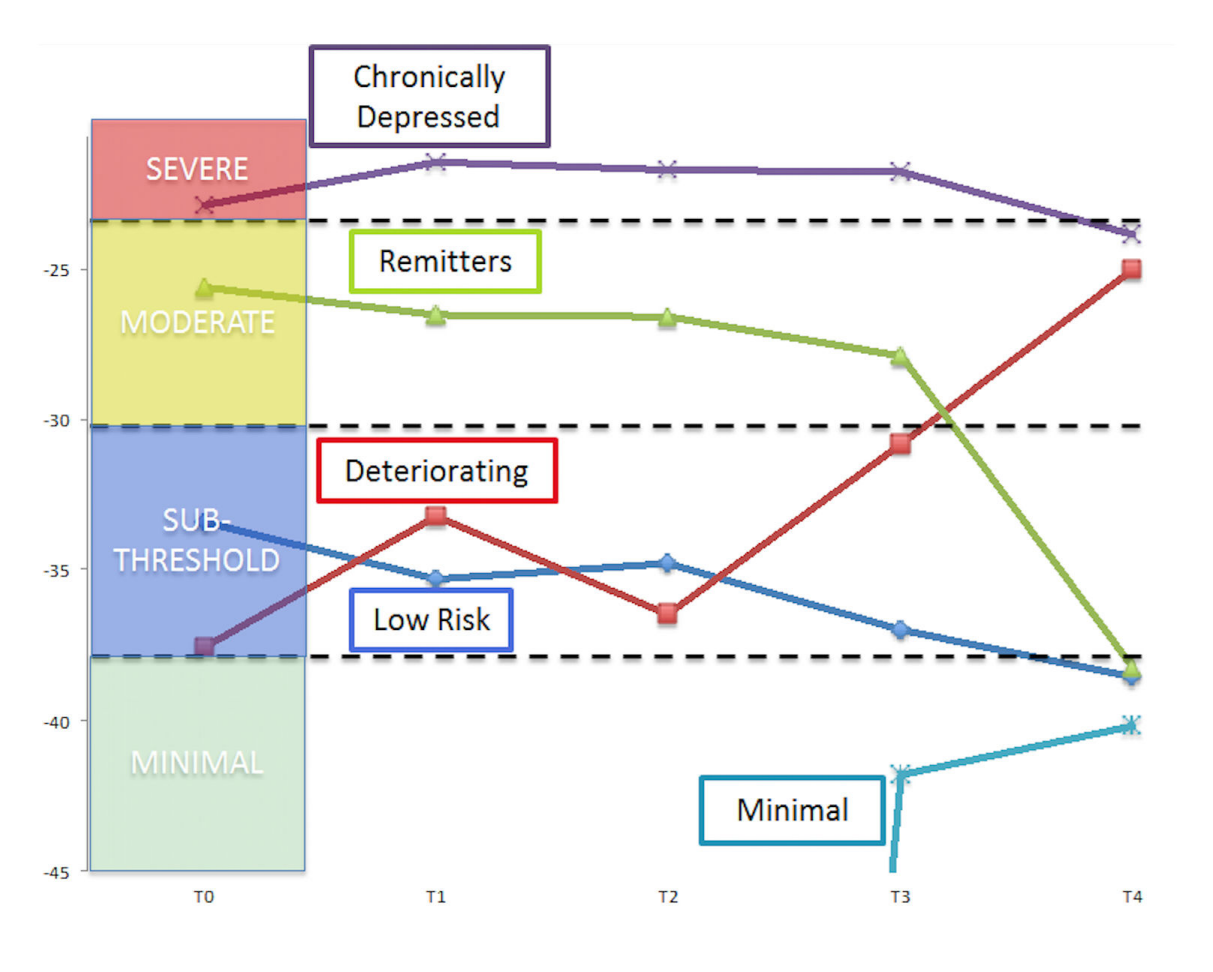
Latent class growth trajectories. The broken line for the minimal class indicates where the threshold drops below the bounds of the figure.

The 5-class solution consisted of a “minimal depression” class (63.66%) whose scores were low and consistent across all time waves, a “low risk” class (18.65%) whose condition remained subthreshold across time, a “deteriorating” class (4.69%) who began at subthreshold but approached severe depression by the end of the study, a “chronic” class (4.96%) who remained highly depressed across the whole study, and a “remitting” class (8.05%) who had moderate depression to start, but crossed the threshold into minimal depression by the end of the follow-up period. Growth parameters are presented in Table 3 and latent trajectory classes are graphically depicted in Figure 1. Note that an ordinal logistic model is used, so the table and figure contain thresholds where the continuous latent variable is cut into each category of depression; the thresholds themselves are not otherwise interpreted.

### Predictors and Consequences of Latent Class Membership

All continuous candidate predictor variables are listed in Table 4, with means and standard deviations broken out by modal class assignment, which refers to the most likely class assignment for each individual. Table 5 lists all categorical candidate predictor variables and relative percentages within each class assignment. Because Tables 4 and 5 are broken out by modal class assignment, the sample proportions of class membership differ slightly from the model based estimates (Table 3,Figure 1).

**Table 4:** Descriptive statistics for continuous predictor variables by modal class assignment.

Continuous variables	Full sample N=22,963	Minimal n^a^=14,676	Low-risk n=4568	Deteriorating n=705	Remitting n=1933	Chronic n=1081
	Mean (SD^b^)	Mean	Mean	Mean	Mean	Mean
Age	45.84 (11.72)	47.09	44.22	41.43	43.72	42.44
Life quality	2.87 (0.79)	3.04	2.69	2.72	2.4	2.09
Health quality	2.63 (0.83)	2.81	2.45	2.47	2.17	1.92
Sleep quality	6.8 (2.14)	7.44	5.98	6.29	5.24	4.61
Sleep hours	7.07 (1.21)	7.24	6.84	6.94	6.67	6.51
Stress	4.04 (1.06)	3.92	4.12	4.14	4.4	4.61
Alcohol use	9.5 (1.34)	9.60	9.39	9.35	9.21	9.16
Physical activity	0.88 (0.32)	0.91	0.86	0.89	0.81	0.80

^a^Class *n* s are based on modal class assignment and differ slightly from the model-estimated class proportions presented in Table 3.

^b^SD: standard deviation.

**Table 5:** Descriptive statistics for categorical predictor variables by modal class assignment.

Categorical variables			Full sample N=22,963	Minimal n=14,676	Low risk n=4568	Deteriorating n=705	Remitting n=1933	Chronic n=1081
			% (n/N)	% (n/N)	% (n/N)	% (n/N)	% (n/N)	% (n/N)
**Depression**								
		Minimal	65.28 (14,959/22,914)	100 (14,638/14,638)	0.02 (1/4560)	45.3 (319/705)	0.05 (1/1932)	0 (0/1079)
		Subthreshold	21.02 (4817/22,914)	0 (0/14,638)	96.97 (4422/4560)	54.8 (386/705)	0.21 (4/1932)	0.46 (5/1079)
		Moderate	10.22 (2341/22,914)	0 (0/14,638)	2.83 (129/4560)	0 (0/705)	91.67 (1771/1932)	40.87 (441/1079)
		Severe	3.48 (797/22,914)	0 (0/14,638)	0.18 (8/4560)	0 (0/705)	8.07 (156/1932)	58.67 (633/1079)
**Gender**								
	Female		66.95 (15,337/22,907)	63.39 (9276/14,634)	70.12 (3196/4558)	74.9 (528/705)	76.85 (1484/1931)	79.05 (853/1079)
	Male		33.05 (7570/22,907)	36.61 (5358/14,634)	29.88 (1362/4558)	25.1 (177/705)	23.15 (447/1931)	20.95 (226/1079)
**Relationship status**								
	Single		12.54 (2917/23,268)	10.76 (1598/14,857)	14.9 (692/4645)	14.6 (104/712)	17.19 (337/1961)	17.02 (186/1093)
	Dating		5.42 (1261/23,268)	4.78 (710/14,857)	6.29 (292/4645)	8.6 (61/712)	6.43 (126/1961)	6.59 (72/1093)
	Married		64.71 (15,056/23,268)	68.45 (10,170/14,857)	59.74 (2775/4645)	59.4 (423/712)	55.74 (1093/1961)	54.44 (595/1093)
	Divorced		14.11 (3284/23,268)	13.02 (1934/14,857)	15.24 (708/4645)	15.3 (109/712)	16.57 (325/1961)	19.03 (208/1093)
	Widowed		1.91 (445/23,268)	1.78 (264/14,857)	2.17 (101/4645)	1.1 (8/712)	2.65 (52/1961)	1.83 (20/1093)
Pregnant			1.33 (305/22,914)	1.24 (181/14,638)	1.69 (77/4560)	1.0 (7/705)	1.45 (28/1932)	1.11 (12/1079)
**Lives with others**								
	Lives alone		12.47 (2863/22,963)	11.6 (1702/14,676)	13.22 (604/4568)	13.6 (96/705)	15.26 (295/1933)	15.36 (166/1081)
	Child 0-2		6.65 (1527/22,963)	6.22 (913/14,676)	7.6 (347/4568)	9.7 (68/705)	6.88 (133/1933)	6.11 (66/1081)
	Child 2-12		26.6 (6109/22,963)	24.75 (3633/14,676)	28.26 (1291/4568)	36.0 (254/705)	29.9 (578/1933)	32.65 (353/1081)
	Child 12-18		20.32 (4666/22,963)	19.34 (2838/14,676)	22.2 (1014/4568)	24.5 (173/705)	20.23 (391/1933)	23.13 (250/1081)
	Adult		74.59 (17,128/22,963)	76.49 (11,225/14,676)	72.66 (3319/4568)	70.5 (497/705)	69.99 (1353/1933)	67.9 (734/1081)
**Weight**								
	Healthy or underweight^a^		33.33 (7654/22,963)	35.45 (5203/14,676)	31.61 (1444/4568)	30.2 (213/705)	27.06 (523/1933)	25.07 (271/1081)
	Overweight		32.27 (7410/22,963)	33.78 (4958/14,676)	30.58 (1397/4568)	28.7 (202/705)	29.85 (577/1933)	25.53 (276/1081)
	Obese		27.84 (6393/22,963)	25.78 (3783/14,676)	29.93 (1367/4568)	31.2 (220/705)	32.07 (620/1933)	37.28 (403/1081)
	Extremely obese		6.56 (1506/22,963)	4.99 (732/14,676)	7.88 (360/4568)	9.9 (70/705)	11.02 (213/1933)	12.12 (131/1081)
**In treatment for depression**			9.01 (2069/22,963)	4.57 (671/14,676)	11.01 (503/4568)	16.3 (115/705)	19.76(382/1933)	36.82 (398/1081)

^a^A minimal proportion of individuals reported underweight body mass index (BMI) in this sample.

After examining the class structure, we were interested in predictors that differentiated the low risk from the deteriorating class, or the remitting from the chronic class. Table 5 presents log odds and odds ratios from the final model, using the minimal depression class as the reference group. It also includes contrasts of interest between the low risk and deteriorating classes, as well as the chronic and remitting classes. The following variables were screened out of the final prediction model because they did not meet our criteria for distinguishing between either set of classes: living with others, weight, relationship status, being pregnant, alcohol use, and physical activity.

**Table 6:** Predictors and consequences of class membership with selected between-class contrasts.

Predictor variables by class		Referent class=minimal			Referent class=Low-risk			Referent class=remitting		
		Log odds	*P*	Odds ratio	Log odds	*P*	Odds ratio	Log odds	*P*	Odds ratio
**Low-risk class**										
	Age	−0.03	<.001	0.97						
	Male	−0.23	<.001	0.79						
	Sleep quality^a^	−0.67	<.001	0.51						
	Sleep hours	−0.10	<.001	0.91						
	Stress^a^	0.20	<.001	1.22						
	Life quality^a^	−0.31	<.001	0.73						
	Health quality^a^	−0.13	<.001	0.88						
	Treatment	0.72	<.001	2.06						
	Distal outcome: WPAI^b^	Mdiff^c^=1.98, *P*<.001								
**Deteriorating class**										
	Age	−0.05	<.001	0.95	−0.02	<.001	0.98			
	Male	−0.49	<.001	0.61	−0.26	.01	0.77			
	Sleep quality^a^	−0.51	<.001	0.6	0.16	.001	1.17			
	Sleep hours	−0.09	.03	0.92	0.01	.77	1.01			
	Stress^a^	0.22	<.001	1.25	0.03	.60	1.03			
	Life quality^a^	−0.30	<.001	0.74	0.01	.91	1.01			
	Health quality^a^	−0.15	.01	0.86	−0.02	.72	0.98			
	Treatment	1.29	<.001	3.64	0.57	<.001	1.77			
	Distal outcome: WPAI	Mdiff=57.25, *P*<.001			Mdiff=55.26, *P*<.001					
**Remitting class**										
	Age	−0.04	<.001	0.96						
	Male	−0.55	<.001	0.58						
	Sleep quality^a^	−0.90	<.001	0.41						
	Sleep hours	−0.12	<.001	0.88						
	Stress^a^	0.43	<.001	1.53						
	Life quality^a^	−0.57	<.001	0.57						
	Health quality^a^	−0.28	<.001	0.76						
	Treatment	1.32	<.001	3.73						
	Distal outcome: WPAI	Mdiff=3.59, *P*<.001								
**Chronic class**										
	Age	−0.06	<.001	0.94				−0.02	<.001	0.98
	Male	−0.58	<.001	0.56				−0.03	.74	0.97
	Sleep quality^a^	−1.12	<.001	0.33				−0.22	<.001	0.81
	Sleep hours	−0.14	<.001	0.87				−0.02	.62	0.98
	Stress^a^	0.55	<.001	1.73				0.12	.001	1.13
	Life quality^a^	−0.94	<.001	0.39				−0.67	<.001	0.69
	Health quality^a^	−0.30	<.001	0.74				−0.03	.67	0.97
	Treatment	2.14	<.001	8.49				0.82	<.001	2.28
	Distal outcome: WPAI	Mdiff=12.70, *P*<.001						Mdiff=9.11, *P*<.001		

^a^To aid interpretation, sleep quality, stress, life quality, and health quality were divided by their baseline standard deviation, meaning the respective odds ratio are commensurate with a 1 standard deviation increase in those predictors.

^b^WPAI: workplace productivity impairment.

^c^Mdiff: mean differences between classes at final time point.

#### Low Risk Versus Deteriorating Class

Individuals who were older or male had lower odds of being in the deteriorating class as compared with the low risk class. Counterintuitively, better sleep quality was associated with greater odds of being in the deteriorating class. Individuals actively receiving treatment for depression or bipolar disorder were more likely to be in the deteriorating class. At the end of follow-up, those in the deteriorating class had much higher productivity impairment (Cohen *d*=3.37) than those in the low risk class.

#### Severe Versus Remitting Class

Age appeared to be a protective factor, with older individuals more likely to be in the remitting class than their younger counterparts. Higher stress levels and lower baseline sleep quality and life quality were all associated with a greater chance of being in the chronic class as compared with the remitting class. Hours of sleep was a significant predictor when examined on its own, but became nonsignificant when including sleep quality in the model. By the end of follow-up, individuals in the remitting class had moderately lower productivity impairment than those in the chronic class (*d*=.55).

#### All Classes Versus Minimal Depression Class

All variables were statistically significant in differentiating the other four classes from the minimal depression class. Individuals engaged in treatment and individuals with higher stress levels were less likely to be in the minimal class than all other classes, whereas increases in age, sleep quality, sleep hours, life quality, and health quality, as well as being male, were associated with an increased likelihood of being in the minimal class as compared with all other classes. Those in the minimal class had lower impairment scores at the end of follow-up than all other classes.

Most notably, however, individuals in the deteriorating group had much greater impairments in functioning as compared with those in the chronic group (*d*=2.72, *P*<.001), despite finishing the study at the same average level of depression.

### Sensitivity and Missing Data Analyses

#### Missing Data Analyses

A binary logistic regression indicated that the following study variables were related to number of assessments over and above the effect of depression: age, health quality, gender, weight, and physical activity. We performed a sensitivity analysis on our latent class structure by regressing latent classes directly on these predictors, in order for the predictors to contribute to maximum likelihood estimates of the class structure and growth trajectories. By including them in the model, maximum likelihood estimation guarantees estimates unbiased by missingness related to these variables. We found the class structure to be substantively the same and class sizes to be nearly identical, with estimated class proportions identical to the second or third decimal places for all classes, indicating the main analyses were not meaningfully biased by missingness mechanisms related to these variables.

#### Sensitivity Analyses

We were surprised by the finding that higher sleep quality was associated with greater odds of being in the deteriorating versus low risk class and speculated that it might be driven by intercept differences, because the low risk class had a marginally larger proportion of individuals in the subthreshold category of depression than did the deteriorating class. We ran a model that controlled for this by regressing intercepts on sleep quality while also including sleep quality as a predictor of class membership. The findings were substantively the same, suggesting that sleep quality differentiated meaningfully between the two classes on both their intercept and slope components.

We conducted several post hoc analyses to examine whether the effect of age on class membership was curvilinear; it was not.

## Discussion

### Trajectories

The first objective of this study was to determine whether longitudinal HRA data would reveal trajectories of depressive symptomatology comparable to those found in research conducted with other populations. The trajectories identified were consistent with existing trends in the literature, but the percentages of participants in each trajectory were somewhat different than those obtained in other studies, a pattern in the previous literature described in the introduction.

As in other studies, most participants did not experience significant depressive symptomatology (“minimal depression”) [7].

Among those manifesting clinical levels of distress, there was a group whose symptomatology continued throughout the study period (“severe”), whereas another segment showed markedly few symptoms over time (“remitters”). These two trajectories replicated chronic and episodic patterns of depressive symptoms observed in studies conducted in clinical settings [5,7,9].

This study revealed that there was a group of HRA participants with subthreshold symptomatology who progressed to greater levels of distress over time (“deteriorating”), mirroring other studies that found a similar pattern of deterioration among individuals with subsyndromal symptoms [4,7,9]. It was noteworthy that although the individuals in the deteriorating group finished the study at the same average level of depression as the chronic group, they had much greater productivity impairment, suggesting that their symptoms had greater impact on daily functioning.

### Predictors

The second objective of the study was to determine whether typical HRA variables would yield predictors associated with the trajectories we observed. The results did identify some key predictors: Some seemed to reflect existing trends in the literature, whereas others did not.

Among those with subthreshold symptoms, individuals who were male and older were less likely to show symptom deterioration, whereas current depression treatment and surprisingly, sleep quality were associated with increased probability of membership in the “deteriorating” class as compared with the “low risk” class. Prior longitudinal research found a U-shaped pattern of depressive symptoms among age cohorts, starting with higher rates among young adults, decreasing during middle age, then increasing in old age [26]. Women reported greater distress in young adulthood, but the gender gap narrowed in old age [26]. Neither of these patterns was observed in our sample.

The fact that participants with subthreshold symptoms who were in treatment were more likely to be members of the “deteriorating” class may reflect several different scenarios, including the possibility that these individuals were experiencing progressively greater difficulty in daily functioning than their peers with comparable levels of symptomatology or had experienced prior depressive episodes, and therefore were more likely to seek treatment. A previous study of participants in the HealthMedia SUCCEED HRA used in this research found that those who were currently receiving depression treatment showed greater functional impairment than those with high levels of depressive symptomatology who were not receiving services [27].

The finding that higher sleep quality was associated with deterioration among participants with subthreshold symptomatology seems counterintuitive, as sleep problems play such a significant role in the clinical course of depression [28,29]. However, various studies indicate that sleeping is a commonly used coping strategy for stress reactions and symptoms of depression and anxiety [30,31] (Multimedia Appendix 1). Those findings make us wonder whether members of the “deteriorating” class had begun to use sleep to cope with prodromal symptoms when they first took the HRA. If this pattern were to appear in similar studies in the future, it may suggest that the use of sleep as a coping strategy by individuals with subthreshold distress might be a leading indicator of more serious depressive symptoms in the future.

Among participants with more significant symptomatology at the outset, those in the “severe” class tended to be younger than their counterparts in the “remitting” class. Lower baseline sleep quality and quality of life, as well as stress level were all predictive of membership in the “severe” class, results that were consistent with prior findings on the connections between those variables and both the severity and clinical course of depression [28,29,32,33].

### Recommendations

The third objective of the study was to determine whether we could make meaningful recommendations for population health management, applicable to HRA participants, based on the predictors we identified. Below we offer several recommendations intended to build on and extend the results of this study:

Nearly a quarter of the population in this study experienced subthreshold depressive symptoms at baseline, and 1 in 5 of them deteriorated during succeeding years, to the point where their level of symptomatology was comparable to the chronic group, experiencing a much greater level of functional impairment at follow-up than those with chronic symptoms. Although this study did find variables that differentiated the “deteriorating” participants from those whose symptoms remained at subthreshold levels, additional research is needed before translating those results into meaningful intervention strategies for these individuals.

There is a need for further data on the convergent validity of depression scales as used in HRAs. Although high level of productivity impairment in the “deteriorating” class supports the belief that these individuals need preventative services, it would be helpful to know more about the clinical accuracy of the depression screening scores. How well do high-risk scores correlate with diagnostic judgments and needs assessments rendered by trained clinicians? Are there additional predictor variables that distinguish individuals in the “deteriorating” and “chronic” classes truly suffering from clinical levels of depressive symptoms from those who are not?

This study used data from an HRA that was administered almost exclusively via desktop computers, mostly before the implementation of the Affordable Care Act (ACA). However, HRAs are increasingly moving to mobile platforms [34] (Multimedia Appendix 2), which may well affect the psychometric properties of existing screening tools, or require shorter instruments such as the Patient Health Questionnaire-2 (PHQ-2). Use of a mobile platform may attract different demographic groups, resulting in younger and more diverse HRA populations. Changes in commercial health plan demographics have already occurred because of ACA implementation [35]: How those changes affect the profile of typical HRA participants will need to be determined.

Results of this study support the notion that mass HRA screenings conducted by health plans and employers can flag depression risk among segments of the population (eg, those who are younger or healthier) who may not be routinely screened in traditional health care settings. Health plans and employers could conduct further research on depressive symptoms among HRA participants:

They can test and refine models for predicting membership in the “chronic” and “deteriorating” classes. For example, the results of this study suggest that asking participants with subthreshold depressive symptoms if they are using sleep as a coping strategy may increase our ability to identify those who symptoms are likely to worsen over time. Below we discuss other variables that might increase predictive power if they were included in future HRAs.

It concerned us that relatively small numbers of participants were receiving treatment for depression, even among the most highly symptomatic individuals. However, as the predictive models are refined, they should provide an opportunity for health plans and employers to test more aggressive outreach strategies (eg, outbound telephone contact) for HRA participants who are likely to belong to the “severe” and “deteriorating” classes (eg, younger participants, and those who manifest poor sleep quality, low quality of life, and/or high stress levels), to increase participation in whatever treatment services are available.

Those who design HRAs may want to include new variables that might provide more insight into the factors influencing class membership, especially those associated with chronic depressive symptoms and exacerbations in subthreshold symptomatology. HRA questions have traditionally focused on modifiable health risks [27], such as physical activity, nutrition, but typically have not assessed psychological variables known to increase vulnerability to depression, such as cognitive style or history of emotional maltreatment [36]. Moreover, HRA items have not usually evaluated protective factors that might reduce risk such as community involvement. However, as HRAs evolve, they are increasingly covering new areas such as mindfulness, vitality, and sense of mission or purpose, that might enable us to gain greater insight into the factors that decrease susceptibility to chronicity or deterioration [34] (Multimedia Appendix 2).

### Limitations

The HRA participants were typically incentivized by their employers to take the assessment. Since participation rates varied widely across employers and health plans using the HRA, self-selection bias undoubtedly plays some role in this sample. The participants tend to be younger and better educated than typical US health plan members [35], and they may well be more interested in their health. As noted earlier, some results were probably affected by the dearth of elderly participants in our sample. However, because millions of people now take HRAs every year, we believe that this continues to be a population worthy of study. As noted above, shifts in technology platforms and health plan demographics will require additional research in order for predictive studies to have any value in the future.

This study utilized only self-report measures, and the results are subject to all the limitations associated with them, especially regarding the depression measure. As discussed earlier, without diagnostic interviews, we have no way of determining the relative probabilities of false positives and false negatives yielded by using this version of the CES-D with this population.

Lastly, as noted above, since the HRA focused extensively on modifiable health risks, we perhaps missed opportunities to gain greater insight into protective factors or psychological variables that increase vulnerability to depressive symptomatology, and help us better understand determinants of class membership.

## Appendix

Multimedia Appendix 1 Highlights, workplace stress and anxiety survey.

Multimedia Appendix 2 Whitepaper: reimagining the health assessment.

## Abbreviations

ACA: Affordable Care Act
BIC: Bayesian information criterion
BMI: body mass index
CAIC: consistent Akaike information criterion
CES-D: Center for Epidemiological Studies Depression Scale
GMM: growth mixture modeling
HRA: health risk assessment
PHQ-2: Patient Health Questionnaire-2
PSS: Perceived Stress Scale
SD: standard deviation
WPAI: work productivity activity impairment
