# Supplementary material for: Trajectories of Depressive Symptoms Among Web-Based Health Risk Assessment Participants
Source: J Med Internet Res. 2017 Mar 31;19(3):e96. doi: 10.2196/jmir.6480 (PMC5392210; doi:10.2196/jmir.6480)
Supplement: Multimedia Appendix 1 [file jmir_v19i3e96_app1.html]

Highlights: Workplace Stress & Anxiety Disorders Survey | Anxiety and Depression Association of America, ADAA


Skip to main content

Member Login

Search


- Consumers
- Professionals

- Conference & Education
  - Conference
    - Why Attend?
      - Justification Tool Kit
      - Conference Attendee Stories
    - Continuing Education
    - Sponsors & Exhibitors
      - Sponsors
      - Exhibitors
    - Conference Committee
    - Future and Past Conferences
    - 2016 Mobile App and Program
    - Media Coverage: ADAA Conference 2016
    - Submissions
    - Hotel and Travel
    - Conference Highlights
    - Registration and Rates
    - Full-Day Workshop With Reid Wilson
  - Professional Education
    - Clinical Fellows
    - Webinars for Mental Health Treatment Providers
      - Recordings: Webinars for Mental Health Treatment Providers
      - Registration Information: Webinars for Mental Health Treatment Providers
    - Professional Development
    - Podcasts for Mental Health Treatment Providers
- Membership
  - Overview
    - Benefits
      - Members Listserv
    - Join as a Professional
    - Join as a Trainee/Postdoctoral Fellow/Resident
    - Join as a Student
    - FAQs
  - Committees
    - Committee Guidelines
    - Scientific Council
  - Special Interest Groups
    - Child & Adolescent Anxiety SIG Peer Consultation
    - OCD & Related Disorders SIG Peer Consultation
  - Professional Opportunities: Jobs and Fellowships
- Journal & Multimedia
  - Announcements
  - Depression and Anxiety
  - Podcasts & Videos
- Resources
  - Clinical Practice Reviews & Teaching Tools
    - Clinical Practice Review: GAD
    - Clinical Practice Review: OCD
    - Clinical Practice Review for Social Anxiety Disorder
    - Treating Anxiety Disorders: Educational Videos
    - Clinical Practice Review for Major Depressive Disorder
  - Meetings & Events
  - Mental Health Apps
  - Announcements
- Awards
  - Alies Muskin Career Development Leadership Program
    - Participants in the Alies Muskin Early Career Development Program
  - Clinician Trainee Awards
    - Recipients of the Clinician Trainee Award
  - Donald F. Klein Early Career Investigator Award
    - Recipients of the Donald F. Klein Early Career Investigator Award
  - Travel Awards
  - Member Recognition Awards
- Consumers

- Consumers

- Member Login
- Professional

## Main navigation

- Understand the Facts 
  - Generalized Anxiety Disorder (GAD)
  - Panic Disorder & Agoraphobia
  - Social Anxiety Disorder
  - Specific Phobias
  - Obsessive-Compulsive Disorder (OCD)
  - Posttraumatic Stress Disorder (PTSD)
  - Depression
  - Bipolar Disorder
  - Suicide and Prevention
  - Stress
  - Hoarding: The Basics
  - Related Illnesses
  - DSM-5: Changes
  - Myth-Conceptions
- Find Help 
  - Find a Therapist
  - Treatment
  - Support Groups
  - Coaching
  - Mental Health Apps
  - Helping Others
  - Self-Help Publications & Materials
- Live and Thrive 
  - Managing Anxiety
  - Personal Stories of Triumph
  - Ask and Learn
  - Children and Teens
  - College Students
  - Women
  - Older Adults
  - Military & Military Families
- Take Action/Support ADAA 
  - Our Partners
  - Spread the Word
  - Donate
  - Ways to Give
  - ADAA Store
  - Speak Out
  - Sign Up to Help: Patient Registries
  - Year-Round Sponsorship
- Blog
- Professionals


## Professionals

- Conference & Education
- Membership
- Journal & Multimedia
- Resources
- Awards
- Consumers

# Highlights: Workplace Stress & Anxiety Disorders Survey

It comes as no surprise that most working Americans experience stress or anxiety in their daily lives. And the Anxiety Disorders Association of America (ADAA) 2006 Stress & Anxiety Disorders Survey backs that up.

A certain amount of stress and anxiety is normal at work as well as at home. However, persistent, excessive, and irrational anxiety that interferes with everyday functioning is often an indication of an anxiety disorder.  Read on for how how stress affects American employees.

#### Stress, Anxiety, and Anxiety Disorders in the Workplace: Snapshot

Self-reporting of anxiety symptoms and prescription medication use are high among America’s employees, but diagnoses of anxiety disorders are dramatically lower.

- 72 percent of people who have daily stress and anxiety say it interferes with their lives at least moderately.
- 40 percent experience persistent stress or excessive anxiety in their daily lives.
- 30 percent with daily stress have taken prescription medication to manage stress, nervousness, emotional problems or lack of sleep.
- 28 percent have had an anxiety or panic attack.
- Only 9 percent have been diagnosed with an anxiety disorder.

#### Other Key Findings

**Workplace Stress and Anxiety Affects Life at Work — and at Home**   
Job stress has professional and personal consequences.  
  
***On the job:*** Employees say stress and anxiety most often impacts their …

- workplace performance (56 percent)
- relationship with coworkers and peers (51 percent)
- quality of work (50 percent)
- relationships with superiors (43 percent)

***During off time:*** More than three-fourths who say stress interferes with their work say it carries over to their personal life, particularly men (83 percent vs. 72 percent for women).  
  
***With spouses, loved ones:*** Seven in 10 of these adults report that workplace stress affects their personal relationships, mainly with their spouses. Men (79 percent) report it affecting personal relationships more than women (61 percent).  
  
***The main culprits*** of work-related stress:

- deadlines (55 percent)
- interpersonal relationships (53 percent)
- staff management (50 percent)
- dealing with issues/problems that arise (49 percent)

**Methods for Managing Workplace Stress**  
Finding relief takes a variety of forms, some healthy and many not.  
  
***Dreaming of a less stressful job?*** The top method of managing high levels of stress at work for both men and women is to sleep more (44 percent total).  
  
***Women and men manage job stress differently:***

- Women are significantly more likely than men to eat more (46 percent vs. 27 percent) and talk to family and friends (44 percent vs. 21 percent) to manage job stress.
- Men are significantly more likely than women to have sex more frequently (19 percent vs. 10 percent) and use illicit drugs (12 percent vs. 2 percent) to manage job stress.

***Common ground exists*** in other ways men and women cope with job stress:

- consuming more caffeine (31 percent)
- smoking (27 percent)
- exercising more frequently (25 percent)
- taking over-the-counter or prescription medication (23 percent)
- consuming more alcoholic beverages (20 percent)

**Employees Fear Repercussions**  
Most employees are not comfortable discussing stress with their employer.  
  
***Tight-lipped workforce:*** Fewer than half (40 percent) employees whose stress interferes with work have talked to their employer about it.   
Here’s why:

- fear their boss would interpret it as lack of interest or unwillingness to do the activity (34 percent)
- fear being labeled “weak” (31 percent)
- fear it would affect promotion opportunities (22 percent)
- fear it would go in their file (22 percent)
- fear being laughed at or not taken seriously (20 percent)

***Help not always on the way:*** Of those who did speak to their employer, four in ten were offered some type of help from their employer, most often a referral to a mental health professional (26 percent) or a relaxation or stress-management class (22 percent).  
  
**Prevalence Among Workers**  
Many employees report suffering from anxiety that is persistent and excessive and affects their ability to function. Yet many fewer reported suffering from an anxiety disorder — a telling inconsistency. Employees whose anxiety interferes with their everyday functioning may be suffering from an anxiety disorder, the most common mental illness in the U.S.  
  
***Anxiety that gets in the way:*** One in four reports persistent stress or excessive anxiety impairing the ability to function in the past six months.  
  
**Chronic anxiety as a way of life?** Four in ten agree that “persistent stress and/or excessive anxiety are a normal part of life,” particularly men (44 percent vs. 36 percent for women).  
  
***Fear of stigma:*** Only one-fourth of those with an anxiety disorder have told their employers. The three-fourths who have not feared…

- their boss would interpret it as lack of interest of unwillingness to do the activity (38 percent).
- it would affect promotion opportunities (34 percent).
- it would go in their file (31 percent).

***Less commonly,*** people with anxiety disorders did not share it with their employers for reasons unrelated to stigma.

- 14 percent didn’t want to produce a doctor’s note.
- 7 percent didn’t think it was their employer’s business.
- 6 percent didn’t think it was necessary.
- 3 percent didn’t want to.

**Disrupting Work and Relationships**   
Employees with an anxiety disorder say it leads to a host of difficulties at work. With more than 18 percent of the adult population suffering from an anxiety disorder, this is likely making much more of an impact on productivity and efficiency at U.S. companies that most employers realize.  
  
***Strained relations:*** Almost half say that it interferes with their relationships with people at work, mainly causing them to avoid social situations (73 percent), become short tempered (53 percent), and avoid participating in meetings (43 percent).  
  
***Symptom triggers:*** Half said their work responsibilities trigger symptoms of their disorder (53 percent), primarily dealing with problems and meeting deadlines. Interpersonal relationships also trigger symptoms (46 percent), as do changes to work situations (37 percent) — such as leaving a job, starting a new one, or getting fired — and staff management (35 percent).  
  
***Trying to cope:*** Employees with anxiety disorders ease their symptoms in a variety of ways, primarily...

- taking over-the-counter or prescription medication (52 percent)
- sleeping more (50 percent)
- eating more (39 percent)
- talking to family or friends (38 percent)
- talking to a medical or mental health professional (37 percent)

***Stark differences between men and women:***

- Men are significantly more likely than women to try to ease their symptoms by having sex more frequently (25 percent vs. 6 percent) and using illicit drugs (11 percent vs. 0 percent).

Share This


End the Suffering:  
Beat Anxiety and Depression

Donate Now

## Get "Triumph," Our E-News

## Therapist Directory

Search our directory of licensed mental health providers who specialize in anxiety disorders, depression, OCD, PTSD, and related disorders.

Find a Therapist

## Webinars

Self-Help Strategies: Webinars to Calm Anxious Minds

## Understand the Facts

- Generalized Anxiety Disorder (GAD)
- Obsessive-Compulsive Disorder (OCD)
- Panic Disorder & Agoraphobia
- Posttraumatic Stress Disorder (PTSD)
- Social Anxiety Disorder
- Specific Phobias

## Conference

- Why Attend?
- Continuing Education

## About ADAA

- Mission & History
- Annual Report
- People
- Our Partners
- Sponsorship, Exhibit, and Advertising Opportunities
- Position Papers
- Donate
- Bylaws

ADAA does not provide psychiatric, psychological, or medical advice, diagnosis, or treatment. See additional information.

## Membership

- Membership Overview
- FAQS
- Standing Committees
- Special Interest Groups
- Member Recognition Awards

## Press Room

- News & Updates
- Fact & Statistics
- Press Releases
- Multimedia

## Quick Links

- Awards Program
- Professional Resources
- ADAA Home
- Find a Therapist

## FAQ

Do I have an anxiety disorder?

What causes anxiety disorders?

How do I find the right health professional?

more FAQs

## Contact ADAA

8701 Georgia Ave., Suite #412  
Silver Spring, MD 20910  
240-485-1001

- How to Be a Sponsor
- Contact ADAA
- Request Publications

## Follow Us

Facebook Twitter RSS Pinterest

## Follow Us

Facebook Twitter YouTube LinkedIn

ADAA is an international nonprofit organization dedicated to the prevention, treatment, and cure of anxiety, depressive, obsessive-compulsive, and trauma-related disorders through education, practice, and research.


Privacy Policy © ADAA, 2010-2016
