# Supplementary material for: Trajectories of Depressive Symptoms Among Web-Based Health Risk Assessment Participants
Source: J Med Internet Res. 2017 Mar 31;19(3):e96. doi: 10.2196/jmir.6480 (PMC5392210; doi:10.2196/jmir.6480)
Supplement: Multimedia Appendix 2 [file jmir_v19i3e96_app2.pdf]

Johnson & Johnson HEALTH & WELLNESS SOLUTIONS

WHITE PAPER

Reimagining the Health Assessment

Johnson & Johnson Health and Wellness Solutions, Inc.

Rich Bedrosian, Ph.D.

Amy Bucher, Ph.D.

Tyler Steben

Scott Rigby, Ph.D.

Bobbi Coluni

Steve Bennett, M.S.

CONTACT

CALL US: 855-206-3815
EMAIL US: JJHWS@ITS.JNJ.COM
VISIT US ONLINE AT:
JJHWS.COM/INSIGHTS/WHITE-PAPERS

©2015 Johnson & Johnson Health and Wellness Solutions, Inc.

WHITE PAPER

Reimagining the Health Assessment

Rich Bedrosian, Ph.D.
Amy Bucher, Ph.D.
Tyler Steben
Scott Rigby, Ph.D.
Bobbi Coluni
Steve Bennett, M.S.

Introduction Health assessments (HAs) are established tools in the world of population health management. Used to gather data on a variety of health risks and behaviors through patient questionnaires, HAs have the potential to provide employers, health plans and researchers with rich data about the prevalence of particular health risks in a population. Over time, repeated administrations of HAs can provide valuable insight into the effects of health interventions and longitudinal trends in population health.

Data from HAs are particularly critical in light of findings that ten modifiable behaviors drive the vast majority of health care costs in the United States (Goetzel et al., 2012). Population-level data can help guide interventions, and personal data may help drive individual behavior change. People who participate in HAs may also make better use of health care services (Sieck & Dembe, 2014).

Challenge

The traditional HA experience has a reputation for being onerous. Question sets are long and require users to access medical information that may not be close at hand. For web-based or mobile HAs, consumers increasingly have low tolerance for poor digital experiences. Additionally, HAs do not always provide users with actionable feedback to help change health-related behaviors in a proven, science-based way.

Opportunity

There is an opportunity to better engage users in effective behavior-change interventions while preserving the integrity of data captured through the HA.

What We Did

Johnson & Johnson Health and Wellness Solutions identified issues with the existing HA model – focusing on the need to improve user engagement while delivering a meaningful data set to economic buyers. We also recognized a need to think beyond traditional health risks to consider other variables influencing health and wellness – essentially transforming the tool from a "health risk assessment" or "HRA" to a health assessment or "HA".

We conducted a series of consumer studies to identify the most critical areas of improvement for increasing user engagement with an HA. Next, we conducted a rigorous statistical validation study to create a streamlined questionnaire designed to

predict health risks. We also incorporated topics like mindfulness and vitality – topics not typically included in an HA but with important implications for health and wellness.

Results/Outcomes

After two rounds of user research, the statistical validation study, and an iterative design process, the result was *My Health Discovery,*™ a fully reimagined health assessment with key features organized around three areas:

• **Optimized question set.** We dramatically reduced the number of questions asked while still providing a meaningful data set to economic buyers. The data set retains crucial constructs for maintaining fidelity to the National Committee for Quality Assurance (NCQA) certification requirements and calculating Health Enhancement Research Organization (HERO) risk scores, both frequent concerns for health plans and employers.

• **Improved user experience.** With a mobile-first design, users can easily engage with the assessment on any device.

• **Enhanced application of behavioral science for engagement.** We leveraged behavior science for engagement and applied motivational design principles, including game mechanics, to offer users elements of choice as they complete the assessment. Discovery-based feedback helps users gain new insights about their health and behaviors and provides positive feedback and encouragement alongside suggestions for mitigating health risks. Tailored and concrete action steps are offered throughout the HA experience and are designed as a natural pathway to additional coaching.

My Health Discovery™ was built for mobile and for use across multiple platforms. It provides an engaging, action-oriented HA experience for users and meaningful population health data for economic buyers.

Conclusion

Johnson & Johnson Health and Wellness Solutions developed *My Health Discovery*™ as a fully reimagined health assessment. With more health plans and employers offering HAs to members, and consumers increasing their expectations around digital experiences, the limitations of traditional HRAs were becoming more evident.
We saw an opportunity to better engage users and promote participation in effective behavior-change interventions while maintaining or improving the integrity of data captured through the HA for the organization providing it.

We believe *My Health Discovery*™ represents a disruptive change in how the market thinks about health risk assessments. *My Health Discovery*™ moves beyond a focus on broad data sets to examine the strategic value of specific, targeted data. This new HA fundamentally improves the user experience and recognizes that, to be effective, an HA must go beyond evaluation to become an engaging, science-based tool that functions as a gateway to improved health behaviors.

THIS WHITE PAPER

In this paper, we will cover the foundational framework upon which *My Health Discovery*™ is built, including perspectives on technology and design that determined product specifications. Then, we will review the research that Johnson & Johnson Health and Wellness Solutions conducted with end users to develop the new HA format. We will also provide an overview of the statistical validation research that was conducted to arrive at a questionnaire that minimizes user burden while providing employers and healthcare organizations rich, meaningful data about their populations. Finally, we will review some of the features and benefits of *My Health Discovery*™ that result from this research.

Health assessments (HAs) have a firm hold in the world of population health management. Used to gather data on a variety of health risks and behaviors through patient questionnaires, HAs have the potential to provide employers, health plans, and researchers with rich data about the prevalence of particular health risks in a population. Over time, repeated administrations of an HA can provide valuable insight into the effects of health interventions and longitudinal trends in population health.

Data from HAs are particularly critical in light of findings that ten modifiable behaviors drive the vast majority of health care costs in the United States (Goetzel et al., 2012). Population-level data can help guide interventions, and personal data may help drive individual behavior change. People who participate in HAs may make better use of health care services (Sieck & Dembe, 2014).

However, HAs also have a reputation for being onerous to users. The question sets may be long and require users to look up medical information that is not close at hand. In one study, nearly 40% of people who declined to take an HA cited time concerns as their main reason (Colkesen et al., 2011). Additionally, unlike digital coaching programs, HAs typically do not provide much actionable feedback to end users. End users therefore do not derive useful information to help change health-related behaviors as a result of the HA experience.

Many of these issues arise from the needs of the HA's economic buyer, whether that is a health plan or an employer. Economic buyers typically want a broad data set on their population, which drives the length of HA question sets. Unfortunately, if end users fail to complete the HA due to the burden of questionnaire length, economic buyers do not receive the data. An HA that users do not complete also misses an opportunity to engage uses in appropriate risk-reducing interventions.

Therefore, it is in the best interest of economic buyers to be judicious about what data are truly necessary to monitor their population and to help inform programs and tools that will help mitigate health risks and add the most value.

At the same time, one effect of U.S. health care reform has been an increasing consumerization of health care. Health plans now have an economic incentive to offer HAs and other wellness programs to their members (Patient Protection and Affordable Care Act, 2010), which means a proliferation of options for end users. As a result, consumers may have lower tolerance for barriers to digital health experiences. For example, users are not likely to be tolerant of digital programs that require a lot of effort to enroll or have non-intuitive navigation. Market forces are requiring companies that administer experiences like HAs to consider the benefits to users and not just data collection.

BUILT FOR HOW PEOPLE USE TECHNOLOGY

My Health Discovery™ was built for mobile-first and for use across multiple platforms, recognizing that users increasingly reach for their smart phones as a primary device. Most HAs have historically been hard copy instruments adapted to a web format, rather than created to take advantage of web capabilities. It has only been in the last few years that some HAs have been developed specifically with an eye to web administration (e.g., Mills et al., 2011).

Now the smart phone and tablet era has arrived. By 2014 the majority of Americans owned smart phones (Pew Research Center, 2014). Over half of Americans surveyed said that their primary device for accessing the web was either a phone or a tablet, not a computer (Chaffey, 2014). There is also a growing segment of people who only access the web through mobile technology (McGrane, 2013). Finally, data show that about 90% of web users transition between multiple devices to complete tasks (Google, 2012). These trends suggest a need to go beyond designing for just the web to designing specifically for the mobile web. Considering *My Health Discovery*™ from a mobile-first mindset allowed the Johnson & Johnson Health and Wellness Solutions team to think creatively about how to format and design HA questions. At the same time, designing for mobile resulted in a more visually interesting web experience for laptop and desktop users as well, helping to better engage users.

A BEHAVIOR CHANGE PERSPECTIVE ON DESIGN

Engaging users in the HA is important for multiple reasons. Not only do economic buyers want users to complete the assessment, but also to provide complete, truthful responses in order to create a high-quality data set. From a public health perspective, the HA also has a higher likelihood of evoking behavior change if it engages users in the actual experience. An HA that helps motivate change, increase user receptivity to feedback, and inform action to mitigate risk is more likely to influence public health than one that does not. The design of the HA is critical in gaining that type of user engagement.

Design is not just the appearance of a product. It is also the underlying psychological framework around which a product's elements are arranged in order to create the user's experience. As a behavior science-based company, Johnson & Johnson Health and Wellness Solutions draws on motivational design to create products that engage users by supporting fundamental psychological needs. In fact, research suggests that digital experiences that support these needs do have a stronger "hook" for users (Przybylski, Rigby, & Ryan, 2010); that is why the motivational design approach is heavily used to create games and gamified programs.

Those fundamental psychological needs, as described by Self-Determination Theory (SDT; Ryan & Deci, 2000) are:

Autonomy. Autonomy refers to the human need for meaningful choice. The idea of meaningful choice is

particularly important; technology applications can offer users hundreds of choices from customizing color schemes to reorganizing elements on a dashboard, but these superficial decisions only minimally support people's sense of autonomy. Autonomy is better fulfilled when users engage with technology in fulfillment of a highly personal goal. This is particularly critical in health behavior change, where many of the specific activities required to achieve outcomes may not be desirable (e.g., eating less of favorite foods, engaging in exercise rather than relaxing after work, or experiencing some medical treatments).

Competence. Competence is the need to learn and grow over time. Humans seek feedback from other people and their environments to benchmark their performance (Bandura, 1971); they become more engaged when that feedback either indicates successful growth or provides useful information for improvement (Senko & Harackiewicz, 2005; Vallerand, 1997). Competence can also be supported when the tasks presented to someone reflect an optimal challenge level, one that stretches abilities without being so easy as to be boring.

Relatedness. Human beings are highly social animals and crave connections with others. These connections may come in the form of actual person-to-person relationships, or they may be more conceptual, as in feeling understood, belonging to the same spiritual community, or perceiving important commonalities between yourself and others. Technology products can promote a sense of relatedness by enabling person-to-person connections or becoming a participant in a relationship with the user. Warm content tone, personalization, and responsiveness to the user's input all help support relatedness (Alison, Alison, Noone, Elntib, & Christiansen, 2013; Sheldon & Filak, 2008; Visser, 2010).

The philosophy behind motivational design shares much in common with gamification. Games hook people by pushing motivational levers – by providing people with a sense of meaningful choice (autonomy), helping them see progress and growth (competence), and connecting them with other, like-minded people (relatedness). In addition to providing support for these needs, a gamified product specifically includes game mechanics, which include points, badges, and leaderboards (Werbach & Hunter, 2012). *My Health Discovery*™ selectively incorporates some game mechanics, including icons, animation, sounds, and levels that support a sense of progress. These mechanics are used only when they naturally support the user activities within the HA, as our design focus is more on fulfilling the underlying psychological needs rather than including such mechanics for their own sake.

At each step in the development process, decisions about content, functionality, and design were compared against the best practices of motivational design. The principles of support for autonomy, competence, and relatedness were thus infused throughout *My Health Discovery*™ to help create an engaging and motivating user experience.

Figure 1: Feedback throughout the data collection process supports relatedness by creating a relationship with users, and autonomy by offering frequent choice points.

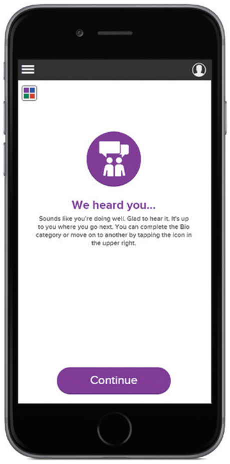

Recognizing that different stakeholder groups and research methods yield different insights into the HA (e.g., Vosbergen et al., 2014), we conducted multiple rounds of research as part of the *My Health Discovery*™ development process.

Design Input: Stakeholder and End User Interviews. As a first step in understanding the key issues to be addressed when reimagining the HA, a third-party team conducted two rounds of investigatory interviews. The first included key internal Johnson & Johnson Health and Wellness Solutions stakeholders representing different functional areas including behavior science, business development, product management, and customer support, as well as existing customers and other external health plan leaders. The intention of these interviews was to understand the business requirements necessary to satisfy economic buyers, as well as the historical challenges with the design and implementation of HAs. The second included end users who were exposed to the existing SUCCEED™ HA for the first time as part of the interview process. These users were queried about their expectations of the HA experience, their thoughts on the design and usability of the existing program, and ideas for improvements.

This initial interview research yielded four design principles to guide the development of the reimagined health assessment. The principles were derived from end-user feedback and aligned with business stakeholder concerns around gathering high-quality data, engaging end users, and improving end users' health over time. The principles are also in alignment with the motivational design principles underlying the product design, especially around supporting relatedness in the way the user and product interact. These principles were:

• ***Show me I can trust you.*** HAs require end users to volunteer highly personal information, from biometric results to health-related behaviors to barriers to change. End users shared a concern about offering such data without understanding who would see it and how it would be used. In particular, end users do not want their health care providers or employers seeing non-de-identified health data. They also expressed a need to understand the origin of any health recommendations made to them, in order to judge their credibility and reliability.

• ***Accurately represent me.*** Users reported feeling like the assessment did not accurately differentiate between their health history and their more recent health behaviors. For example, one user felt that his health behaviors, which included a nutritious diet and regular exercise, were de-emphasized in feedback relative to his high cholesterol, which he attributed to family history. He felt de-motivated by the focus on factors he felt were out of his control. Another user had recently lost a great deal of weight but remained outside of a recommended weight range; he wanted a way to enter his historical data so that the assessment could recognize his weight loss accomplishments or understand the progress he had made.

Figure 2: Question design takes advantage of the mobile format for visual interest and interactivity.

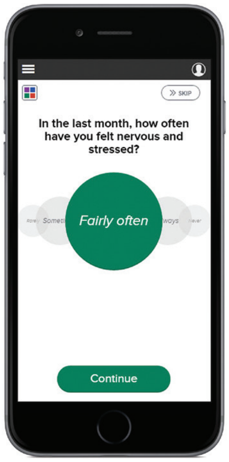

• ***Engage with me.*** Users wanted an HA experience that was interesting and interactive, beyond a typical multiple-choice questionnaire. Engagement encompassed multiple factors. One consideration was making the most of the technology available; users wanted the capabilities and conveniences of mobile devices reflected in the assessment design. Another factor was stating questions in a way that was relevant and comprehensible to the user. One user, for example, disliked a question about waist circumference because finding the answer required him to seek out a tape measure and use it. A second common challenge was estimating food serving sizes, since people rarely think about their meals in the same terms used in most HA question sets. Such activities may be a barrier to assessment completion, so alternative ways of gaining information would be helpful.

• ***Encourage me to act.*** Users wanted to leave the assessment experience with an accurate understanding of their health risks and ideas of steps they could take to mitigate them. They were dissatisfied with feedback that overemphasized factors they felt outside of their control (as with the user with the family history of high cholesterol), and preferred feedback that provided a clear avenue for action.

These four principles were then used to guide the development of initial HA concepts and wireframes, which were then tested with additional end users.

Wireframe and Concept Testing. Once prototypes of *My Health Discovery*™ were developed, including new question formats that take advantage of the touch-screen capabilities of mobile devices, end-user testing was conducted with a group of participants demographically similar to the core user base seen in our health plan customers. The participants each completed a 45-minute, one-on-one in-depth interview (IDI) with a moderator, during which they interacted with a prototype version of the new HA as well as the existing SUCCEED SNAPSHOT™ HA (a condensed version of the full-length SUCCEED™ instrument), which served as a comparison.

The order in which participants were exposed to the assessments was counterbalanced, with half seeing the new mobile HA first and the other half seeing the classic SUCCEED SNAPSHOT™ HA first. Regardless of which instrument they saw first, most users agreed that the new mobile design was more flexible and accessible and said they preferred it.

One key finding from this user testing was that users appreciate a balance between modern and traditional design. Including the typical HA questions orients the user to the experience and set expectations, while enhanced functionality on other questions provides a "wow" factor and increases engagement. Users also expressed a need for a design compatible with mobile browsing, as the smart phone is often the first technology they use to interact with a website. At the same time, they asked that functionality be simple and clean, so they did not need to spend time puzzling out how to use the program but could focus instead on the content of the assessment.

Another finding was that even though users found the new mobile HA engaging, they were not willing to download a native app devoted specifically to an HA. Native apps draw on phone battery life and storage, and users guard this real estate for apps they use frequently, as opposed to an HA they may access only a few times per year. This was one of many considerations that led to *My Health Discovery*™ being developed with responsive design for access through a web browser. Users are not required to download anything to access the tool, and are able to seamlessly move between devices to continue accessing *My Health*

Discovery™ and the associated suite of digital health coaching programs. We believe this design choice mitigates possible barriers to assessment use associated with native apps.

Participants responded favorably to the positive language and encouraging tone in both assessments. Similar to the users from the first round of testing, these participants wanted to hear positive feedback for their accomplishments and encouragement in areas for development. Users specifically called out a sense of not being alone in their health improvement goals as a feature they liked.

Finally, participants provided numerous suggestions about optimizing the navigational experience within the HA, as well as design improvements. These ideas were carried forward into the final visual and architectural design.

INTELLIGENT STREAMLINED QUESTION SET: STATISTICAL VALIDATION RESEARCH

As part of reimagining the HA, we conducted rigorous statistical testing to streamline and validate the question set used. Our goal for this work was to minimize user burden by asking only the most relevant and meaningful questions for each key health risk domain, ensuring relevant and high-quality data for economic buyers while improving the end-user experience. Statistical validation provides confidence that the questions included both measure the intended constructs and adequately predict related outcomes. In essence, the statistical validation allowed our team to select the minimum question sets that would accurately measure the health risks of interest to economic buyers and end users.

Generate candidate constructs and items. To that end, we first created an inventory of the critical health risk domains that *My Health Discovery*™ should include given the concerns of economic buyers and the need to identify risk and motivate behavior change for end users. For each domain area identified, we assembled a set of questions that could measure it, including items from the

Domain	Final Question Set
Basic demographics	1
Screenings & immunizations	4
Biometrics	8
Self-perceived health	2
Personal health history	1
Eating behaviors	3
Alcohol consumption	3
Smoking & tobacco use	3
Physical activity	4
Movement	3
Pain	2
Sleep	2
Productivity	3
Subjective vitality	8
Stress coping	4
Depression	3
Mindfulness	5
Values	4
**TOTAL ITEMS**	**63**

existing SUCCEED™ HA, validated items from published scales, and face-valid equivalents designed by the Johnson & Johnson Health and Wellness Solutions team to take advantage of mobile capabilities. We included several new and innovative areas (or "domains") to reflect our philosophy toward behavior change and motivation. Specifically, we added items around vitality, mindfulness, motivational quality, and values that we believe are necessary to understand in order to coach people through long-lasting behavior change. This comprehensive list of domains and corresponding items became the central instrument for the validation study.

Select a representative study sample. For the statistical validation study, we recruited a sample of approximately 1,700 people who were representative of the overall U.S. population in terms of demographic composition. They ranged in age from 18 to 80 years old, were 50% male, and came from all 50 U.S. states. Choosing a sample that is similar to the overall U.S. population maximizes the generalizability of the research results to our book-of-business customers.

Validate selected constructs. Once the participant sample completed the candidate construct instrument, we analyzed the results using principal components analysis (PCA), confirmatory factor analysis (FA), and related methods. These analyses are designed to assess whether a given item is accurately assessing the right construct. For example, if there are ten questions all intended to measure physical activity, these analytical tools can help determine which ones actually measure someone's exercise habits and which ones are actually assessing related ideas (e.g., motivation to exercise or access to a gym).

The results of these analyses allowed us to select the candidate items for each domain that were measuring the constructs as intended. We retained the subset of questions for each domain that were most strongly correlated with the construct in the data set and were most consistent in their measurement of the desired construct.

Maximize predictive value. In reducing the candidate item list to select the final instrument, we also paid attention to how well each item predicted variation in outcome variables. One of the ultimate goals of any HA tool is to accurately predict health risks and outcomes, as well as guide economic buyer decisions about population-level interventions to offer. Accordingly, we prioritized questionnaire items with a higher predictive validity for meaningful outcomes related to key business metrics such as health care utilization and productivity.

After the two rounds of user research, the statistical validation study, and an iterative design process, the result was a reimagined HA with several key features. These features are organized around three areas:

- Optimized question set
- Improved user experience
- Enhanced application of behavioral science for engagement

Optimized question set. First, *My Health Discovery*™ boasts a dramatically reduced question set. The classic HealthMedia® SUCCEED™ HA, the most robust instrument Johnson & Johnson Health and Wellness Solutions offers, includes a maximum question set of nearly 270 items. *My Health Discovery*™ has a maximum of 63 questions; most users will see a subset of those depending on the skip logic triggered by their responses (e.g., smoking status, pregnancy, etc). The reduced question set improves the user experience by reducing the amount of time needed to complete the instrument.

At the same time, we are able to provide a **meaningful data set** to economic buyers as a result of the statistical validation research, even with an overall reduced number of items. The fact that the questions that were retained were shown to be valid measurements of the intended constructs and significant predictors of outcomes of interest means that we were able to maintain the data rigor desired by customers even while improving the end-user experience. The data set also retains crucial constructs for maintaining fidelity to NCQA certification requirements and calculating HERO risk scores, both frequent concerns for health plans and employers.

Another important feature of *My Health Discovery*™ is the **inclusion of items and domains that go beyond the typical HA.** In addition to standard constructs related to health risks, we also assess

Figure 3: My Health Discovery categorizes questions as belonging to one of four areas, and allows users to choose the order and depth in which they explore each.

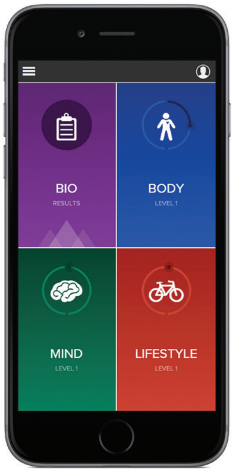

vitality, mindfulness, and values. These topics have increasingly become the focus of behavioral science research on how mental or psychological wellness can influence physical health and well-being (e.g., Boyle, Barnes, Buchman, & Bennett, 2009; Consedine & Butler, 2014; Niemiec, Ryan, Patrick, Deci, & Williams, 2010), so including them in the assessment also recognizes and incorporates recent advances in understanding health. In general, our behavior-change philosophy emphasizes shifting motivational quality toward more intrinsic forms of motivation driven by values and purpose. We include questions to assess users' motivational state and core goals. This information then supports our ability to create a user experience that supports fundamental psychological needs like autonomy, competence, and relatedness.

Improved user experience. With the focus on mobile-first design, we were able to create an assessment experience that allows users to easily engage with the program on any device. Responsive design means that the visual elements on the screen re-organize and re-size automatically to display correctly on a smart phone, tablet, or computer. At the same time, the design takes advantage of the touch screen capabilities of

mobile devices (these questions adjust to be mouse-appropriate for computer users).

The overall design is clean and simple. Feedback from users who interacted with the prototype was used to improve the original navigational flow. The design is colorful and uses animated graphics to pique visual interest and guide the user's interaction with the screen. Visuals also help reinforce message content, consistent with research demonstrating that images can enhance intention to take action on health risk information (Hollands & Marteau, 2013).

All content, including the questions, was also written to optimize the mobile experience. Questions and feedback are short and easy to understand. This style of content also accommodates users who have lower reading levels or simply dislike reading long blocks of text.

Figure 4: Feedback at the end of each level provides a sense of progression and accomplishment, reinforces positive health areas, and indicates opportunities for action.

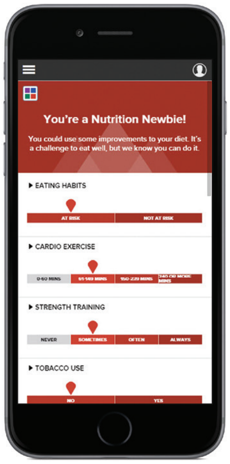

Finally, an important finding from the prototype testing was that users valued the positive, encouraging tone in HA feedback. Accordingly, *My Health Discovery*™ maintains a supportive tone rather than an emphasis on negative feedback. While the feedback alerts users to specific health risks they may have, it does so in a solution-focused manner that emphasizes the user's strengths and ability to make changes.

Enhanced application of behavioral science for engagement. User engagement in the HA is important for at least two reasons. It provides more and better-quality data to the economic buyer, and it increases the chances that the user will receive the information or feedback needed to help mitigate health risks. For the *My Health Discovery*™ experience, we implemented a number of features to improve user engagement with an eye both to completing the instrument and going on to engage in further interventions.

Figure 5: Users are provided with concrete action steps to take to help improve their health risks in each area. This supports competence, builds self-efficacy, and provides a natural bridge to future health coaching interventions.

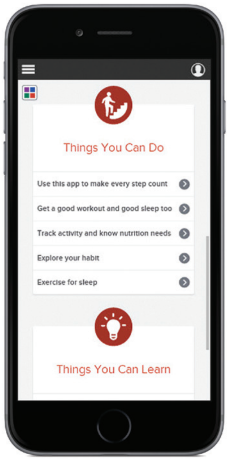

First, users are provided with elements of choice as to how they want to complete the assessment. The question set was divided among four categories: Bio (demographics and biometrics), Body (health history and medical treatments), Lifestyle (exercise, alcohol, and tobacco use), and Mind (mental health, stress management, and mindfulness). Users can choose to dive into the categories in any order they wish. Offering users choice about how to tackle the HA questions supports their sense of autonomy, one of the three basic levers of motivation (Ryan & Deci, 2000). Supporting autonomy has been shown to enhance

engagement with digital experiences (Ryan, Rigby, & Przybylski, 2006). Importantly, because the HA includes calls to action, the choice offered within the design is not merely about sequencing the experience, but about a more fundamental decision on prioritizing health behaviors.

Then, within each category, there are two levels of questions. Level 1 is the most basic questions representing the minimal information needed to provide feedback, with Level 2 being deeper-dive items that can be used for more nuanced and tailored feedback. The level design was chosen in order to provide opportunities for feedback throughout *My Health Discovery*™, rather than in a single block at the end of the questionnaire. Some users may stop the questionnaire midway and never see feedback that may be useful, while also depriving the economic buyer of having that data in their population report. This new design ensures that users receive feedback steadily throughout the questionnaire experience and their data are stored at the end of each level, not when the full questionnaire is submitted. This ongoing feedback supports users' sense of competence by signaling progress through the HA experience as well as reflecting back their "performance" in managing health risks.

The type of feedback offered at the conclusion of each level is discovery-based. That means it is designed to help users gain new insight about their own health and behaviors. The user testing revealed that people feel discouraged when they receive overwhelmingly negative feedback about their health, especially if it is related to factors they cannot control (family history) or morally-tinged behaviors (e.g., overeating). In fact, digital engagement is enhanced when the experience allows users to close the gap between their actual and ideal selves (Przybylski et al., 2011); positive feedback that also indicates avenues for improvement can help accomplish that.

Accordingly, the discovery-based feedback was designed to always provide some positive feedback and encouragement alongside suggestions for mitigating health risks. Moreover, the feedback is designed to highlight relationships between different health variables for users to help deepen their understanding of their health. For example, the body-mind connection is reinforced for users when they learn that their mindfulness can benefit attempts to change health behaviors, or how visualization techniques can reduce pain.

Some game mechanics, as appropriate, were also included in *My Health Discovery*™ to help support user engagement. One example is presenting the discovery-based feedback at the conclusion of each level of questions. This design incorporates the game mechanics of **achievements, levels,** and **progression.** Colors, images, and sounds are used to reinforce the sense of progress through levels and achievement at completion. *My Health Discovery*™ also enables economic customers to implement some of their own game mechanics, particularly related to **rewards** and **bonuses** via their incentives structure.

Historically, HAs are not considered interventions because their role is to collect data on health risks, not prescribe changes to mitigate them. Those HAs that do provide feedback to users typically offer a descriptive portrait of the user's overall health, with an emphasis on rank-ordered risk areas. HAs that do not provide feedback also miss an opportunity to influence users' behavior in a meaningful way. As part of a motivating and discovery-based process, we wanted to empower users to take action on their health risks, not just learn about them. Helping people to take action is also a way to support a sense of competence and build self-efficacy. Therefore, *My Health Discovery*™ offers concrete action steps throughout the experience that the user can try to improve their health risks and adopt healthy new behaviors.

The action steps were designed to align with what Fogg and Hreha (2010) call "dot" activities; that is, they are discrete, one-time activities that would be appropriate on a to-do list and can be checked off as complete in a relatively short time frame. For example, a user with a chronic condition requiring medication might be given an action step to transfer all of her prescriptions to a single pharmacy to streamline the refill process and gain visibility to potential interactions. Dot action steps can also be strung together by the user into a repeating pattern Fogg and Hreha (2010) call a "path" or a "span"; for example, someone learning to be more physically active may take a post-dinner walk every day for a defined period of time.

Finally, *My Health Discovery*™ was designed as a natural pathway to further interventions to improve health-related behaviors. Recognizing that many customers will implement the HA alongside other digital health coaching products, we wanted to create a seamless flow between the initial assessment experience and ongoing coaching. The incremental feedback and action steps included in *My Health Discovery*™ transition the user to our skills-based coaching model, which also uses action steps on a to-do list to organize behavior change. Based on findings that many HA users already take some action on their risks following feedback (e.g., Sieck & Dembe, 2014), we felt it critical to formalize an opportunity to help improve health risks as a result of the risk appraisal.

THE HEALTH ASSESSMENT OF THE FUTURE

We believe *My Health Discovery*™ represents a disruptive change in how the market thinks about assessing health risks. We are moving beyond a focus on the broad data set to the strategic value of the specific, targeted data collected. At the same time, we are focusing heavily on user experience in recognition that an effective HA must go beyond being just an evaluation instrument to being an engagement tool and gateway into improved health behavior.

We also recognize that technology and health care will continue to evolve in the United States and worldwide. Therefore we do not see *My Health Discovery*™ as an end state, but rather a milestone in a continuous product evolution. Our post-launch plans include ongoing outcomes and user experience research to continue to refine the question set, design, and functionality of the solution so that it best bridges the needs of economic buyers and end users.

REFERENCES

Alison, L. J., Alison, E., Noone, G., Elntib, S., & Christiansen, P. (2013). Why tough tactics fail and rapport gets results: Observing rapport-based interpersonal techniques (ORBIT) to generate useful information from terrorists. *Psychology, Public Policy, and Law, 19*(4), 411-431.

Bandura, A. (1971). *Social learning theory.* New York: General Learning Press.

Boyle, P. A., Barnes, L. L., Buchman, A. S., & Bennett, D. A. (2009). Purpose in life is associated with mortality among community-dwelling older persons. *Psychosomatic Medicine, 71*(5), 574.

Chaffey, D. (2014, August 11). How important are digital communications in 2014? Retrieved from http://www.smartinsights.com/marketplace-analysis/customer-analysis/digital-communications-use -statistics-2014/

Colkesen, E. B., Kraaijenhagen, R. A., Frings-Dresen, M. H. W., Sluiter, J. K., van Kalken, C. K., Tijssen, J. G., & Peters, R. J. (2011). Participation in a workplace web-based health risk assessment program. *Occupational Medicine, 61*(8), 586-589.

Consedine, N. S., & Butler, H. F. (2014). Mindfulness, health symptoms and healthcare utilization: Active facets and possible affective mediators. *Psychology, Health & Medicine, 19*(4), 392-401.

Fogg, B. J., & Hreha, J. (2010). Behavior wizard: a method for matching target behaviors with solutions. *In Persuasive Technology* (pp. 117-131). Springer Berlin Heidelberg.

Goetzel, R. Z., Pei, X., Tabrizi, M. J., Henke, R. M., Kowlessar, N., Nelson, C. F., & Metz, R. D. (2012). Ten modifiable health risk factors are linked to more than one-fifth of employer-employee health care spending. *Health Affairs, 31*(11), 2474-2484.

Google (2012, August). The new multi-screen world: Understanding cross-platform user behavior. Retrieved from https://ssl.gstatic.com/think/docs/the-new-multi-screen-world-study_research-studies.pdf

Hollands, G. J., & Marteau, T. M. (2013). The impact of using visual images of the body within a personalized health risk assessment: An experimental study. *British Journal of Health Psychology, 18*(2), 263-278.

McGrane, K. (2013, May 28). The rise of the mobile-only user. *Harvard Business Review.* Retrieved from https://hbr.org/2013/05/the-rise-of-the-mobile-only-us/

Mills, P. R., Masloski, W. S., Bashaw, C. M., Butler, J. R., Hillstrom, M. E., & Zimmerman, E. M. (2011). Design, development and validation of the Redbrick Health Assessment: a questionnaire-based study. *JRSM Short Reports, 2*(9), 71.

Niemiec, C. P., Ryan, R. M., Patrick, H., Deci, E. L., & Williams, G. C. (2010). The energization of health-behavior change: Examining the associations among autonomous self-regulation, subjective vitality, depressive symptoms, and tobacco abstinence. *The Journal of Positive Psychology, 5,* 122-138.

Patient Protection and Affordable Care Act, 42 U.S.C. § 18001 et seq. (2010).

Pew Research Center (2014). The web at 25 in the U.S. Available at: http://www.pewinternet.org/2014/02/25/the-web-at-25-in-the-u-s

Przybylski, A. K., Rigby, C. S., & Ryan, R. M. (2010). A motivational model of video game engagement. *Review of General Psychology, 14*(2), 154.

REFERENCES

Przybylski, A. K., Weinstein, N., Murayama, K., Lynch, M. F., & Ryan, R. M. (2011). The Ideal Self at Play: The Appeal of Video Games That Let You Be All You Can Be. *Psychological Science, 23(1),* 69–76.

Ryan, R. M., & Deci, E. L. (2000). Self-determination theory and the facilitation of intrinsic motivation, social development, and well-being. *American Psychologist, 55(1),* 68.

Ryan, R. M., Rigby, C. S., & Przybylski, A. (2006). The motivational pull of video games: A self-determination theory approach. *Motivation and Emotion, 30(4),* 344-360.

Senko, C., & Harackiewicz, J. M. (2005). Regulation of Achievement Goals: The Role of Competence Feedback. *Journal of Educational Psychology, 97*(3), 320.

Sheldon, K. M., & Filak, V. (2008). Manipulating autonomy, competence, and relatedness support in a game learning context: New evidence that all three needs matter. *British Journal of Social Psychology, 47*(2), 267-283.

Sieck, C. J., & Dembe, A. E. (2014). A 3-Year Assessment of the Effects of a Self-Administered Health Risk Assessment on Health Care Utilization, Costs, and Health Risks. *Journal of Occupational and Environmental Medicine, 56*(12), 1284-1290.

Vallerand, R. J. (1997). Toward a hierarchical model of intrinsic and extrinsic motivation. *Advances in Experimental Social Psychology, 29,* 271-360. http://dx.doi.org/10.1016/S0065-2601(08)60019-2

Visser, C. (2010). Self-determination theory meets solution-focused change: Autonomy, competence and relatedness support in action. *InterAction-The Journal of Solution Focus in Organisations, 2*(1), 7-26.

Vosbergen, S., Mahieu, G. R., Laan, E. K., Kraaijenhagen, R. A., Jaspers, M. W., & Peek, N. (2014). Evaluating

a web-based health risk assessment with tailored feedback: what does an expert focus group yield compared to a web-based end-user survey? *Journal of Medical Internet Research, 16*(1).

Werbach, K., & Hunter, D. (2012). *For the win: How game thinking can revolutionize your business.* Wharton Digital Press.

*Links to other parties' websites are provided solely for convenience. Johnson & Johnson Health and Wellness, Inc. is not responsible for the content of such websites, and users are solely responsible for compliance with any terms of use thereon. In addition, such links to third-party sites does not imply an endorsement of any third party or any website or the products or services provided by any third party.

Johnson & Johnson

HEALTH & WELLNESS SOLUTIONS
